# Prevalence of refractive errors and color vision deficiency in a population of industry-workers in Abhar, Iran

**DOI:** 10.1097/MD.0000000000027758

**Published:** 2021-11-19

**Authors:** Masoumeh Ahadi, Afsaneh Ebrahimi, Saeed Rahmani, Alireza Akbarzadeh Baghban

**Affiliations:** a Student Research Committee, Shahid Beheshti University of Medical Sciences, Tehran, Iran; b Eye Clinic in Omid Hospital, Abhar, Iran; c Department of Optometry, School of Rehabilitation Sciences, Shahid Beheshti University of Medical Sciences, Tehran, Iran; d Proteomics Research Center, Department of Biostatistics, and School of Allied Medical Sciences, Shahid Beheshti University of Medical Sciences, Tehran, Iran.

**Keywords:** color vision deficiency, prevalence, refractive errors

## Abstract

Visual impairment due to refractive errors and color vision deficiency (CVD) can affect the visual abilities of workers in workplace. Identifying the prevalence of common visual problems helps us to prevent and treat occupational ocular problems.

This study was conducted on 2600 males referring from companies for a routine medical exam to Occupational Medicine Center. In all subjects, visual acuity and refraction were measured. Assessment of color vision was performed by Ishihara color test. In present study, right eyes of subjects were selected to statistical analysis.

The mean spherical equivalent was –0.19 ± 1.39 diopter with a range of –11.00 to +10.00 diopter. Whereas 71% of persons were emmetropic, 20% and 9% of them were myopic and hypermetropic, respectively. From a total subjects, 164 of them had CVD with prevalence of color blindness of 6.3%. In comparison with normal subjects, CVD had no significant effect on refractive findings of our subjects (*P* > .05).

Our data present the prevalence of refractive errors and color blindness among Iranian industry-workers. Compared with other studies, our subjects have a lower prevalence of refractive errors, and similar rate of prevalence of color blindness.

## 1 Introduction

Routine eye examination to meet a required vision is necessary for workers. Good vision is an essential for many careers and visual component has an important role in most job functions.^[1,2]^ Refractive errors and color blindness are common vision disorders that would be a potential source for visual-related problems among workers in the workplace. Visual impairment due to these disorders can and do alter the visual abilities of workers.^[3–5]^

Refractive errors are the most common type of visual impairment in the world and the second treatable cause of blindness.^[6]^ Surprisingly, 20% of blindness cases worldwide and 50% of vision loss cases worldwide are due to uncorrected refractive errors.^[7,8]^ Refractive errors lead to major health problems such as pathologic complications, economic costs, decreased vision-related quality of life, and increased vision-related tasks problems.^[9,10]^ Prevalence of refractive errors have been reported various rates between 6% to 45% in several studies.^[11–14]^ It estimates more than 22% of people in the world with myopia. In East Asian countries, myopic prevalence increased to 70% to 80% and in Western countries, it reached to 25% to 40%.^[15–17]^

Color vision deficiency (CVD) or color blindness is the inability to distinguish certain shades of color.^[18]^ Color blindness can be classified as congenital or acquired (due to diseases or drugs). Congenital disease affects predominantly males.^[19]^ In several studies, 1% to 8% of men and 0.4% to 3% of women have CVD.^[20–23]^ Even though the color blindness does not cause a considerable problem in common life, but it sometimes causes the life or financial problems in some jobs. Most of the patients do not know about their color visual deficiency and find about it when they go to work in visual examinations.^[24,25]^

So far, several studies have been conducted on the prevalence of refractive errors and CVD, especially in students, but no studies have been performed on workers in the workplace. In this study, we attempted to determine the prevalence of refractive errors and color blindness in a population of industry-workers in Abhar, Iran.

## 2 Methods

### 2.1 Ethics statement

This cross-sectional study, after approval by the Human Ethics Committee of Shahid Beheshti University of Medical Sciences (number IR.SBMU.RETECH.REC.1398.255) performed in industry-workers coming to the Occupational Medicine Center in Abhar, Iran. Under the principles of Helsinki declaration and detailed explanation of the research, the consent form was received from all participants.

### 2.2 Population and samples

This cross-sectional study was conducted on industry-workers in Abhar, Iran in 2020. Abhar is one of the industrial cities of Iran and have many companies in different industries (mining, machinery, steel, chemical, basic metals, sanitary detergent, textile, food, and polymer). There are more than 200 small to large industrial units with more than 13,000 workers (20% of people's employment). Our subjects were industry-workers referred from their companies for a routine medical exam. More than 90% of workers in industrial units were male. They were from rural and urban areas. Since in the study by Khalaj et al,^[20]^ the prevalence of CVD in men and women in Iran was 4.10% and 0.7%, respectively, so we studied male workers. According to confidence level of 99% and prevalence estimate of 4.10% by Khalaj et al^[20]^ and estimation error of 0.01, the number of samples were calculated 2600.

### 2.3 Examinations

In all participants, visual acuity was measured by Snellen chart. Objective refraction was performed by autorefractometer (Topcon Medical Systems, KR800) and refined by an experienced optometrist. When ocular refraction get outside of normal state (emmetropia), it leads to refractive error and blurred vision. Spherical equivalent (SE) (the numerical sum of the sphere power with half of the cylinder power) was considered as refractive errors of subjects. According to SE, 3 refractive conditions are defined: emmetropic refraction (SE from –0.50 to +0.5 diopter [D]), hyperopic refraction (SE more than +0.50 D), and myopic refraction (SE less than –0.50 D). Assessment of color vision was performed by Ishihara color test (2000, 24 plates). Ishihara pseudoisochromatic plates test is the most common test for routine color vision screening in the world. Sensitivity and specificity of Ishihara color test are 96% and 98.5% respectively with good reliability.^[25]^ We explained the procedure to our subjects and asked them to read the plates with both eyes with spectacle correction if there is 1 under standard illumination (200–250 luxes). After recording responses, state of color vision of participants were determined. In this study, right eyes of subjects were selected to statistical analysis.

### 2.4 Statistics

Our data were analyzed by IBM SPSS Statistics for Windows, (version 21.0, IBM Corp., Armonk, NY). After assessment of normal distribution of data with the Shapiro-Wilk test, we used chi-square, Mann-Whitney, Kruskal-Wallis, and Spearman correlation tests.

## 3 Results

Two thousand six hundred men with mean age of 29.17 ± 5.74 (range 18–46) years were participated in present study. Best corrected visual acuity was 20/30 or better in all participants. The mean SE was –0.19 ± 1.39 D with a range –11.00 to +10.00 D (Table 1). Whereas 1843 persons (71%) were emmetropic, 533 (20%) subjects and 223 (9%) of them had refraction of myopia and hypermetropia respectively. We divided subjects into 5 subgroups according to magnitude of refractive errors including emmetropia, low and high myopia, low and high hypermetropia (Table 2). Four hundred twenty-nine persons (16%) and 104 persons (4%) had low and high myopia, respectively. Also, 207 subjects (8%) and 16 (1%) of them had low and high hypermetropia, respectively.

**Table 1:** Prevalence of refractive errors according to type of objective refraction.

Type of refraction	Mean refraction (D)
Emmetroia = 1843 (71%)	0.20 ± 0.29
Myopia = 533 (20%)	–2.16 ± 1.66
Hyperopia = 223 (9%)	1.38 ± 1.34
Total = 2600	–0.18 ± 1.38

Diopter: D.

**Table 2:** Distribution of refractive errors magnitude.

Refraction	Mean refractive errors (D)
Emmetroia (–0.5–+0.5 D) = 1843 persons (71%)	+0.19 ± 0.28
Low myopia (–3.0– –0.5 D) = 429 persons (16%)	–1.52 ± 0.66
High myopia (<–3.0 D) = 104 persons (4%)	–4.91 ± 1.85
Low hyperopia (0.5–+3.0 D) = 207 persons (8%)	+1.05 ± 0.49
High hyperopia (>+3.0 D) = 16 persons (1%)	+5.19 ± 2.56

D = diopter.

According to age, we divided our subjects into 3 age groups: under 30, between 30 and 40, and over 40 (Table 3). In the first group (under 30), the prevalence of myopia and hypermetropia was 19% and 8%, respectively. And in the second group (between 30 and 40), the prevalence of myopia and hypermetropia was 24% and 8%, respectively. But in the third group (over 40), the prevalence of myopia and hypermetropia was 15% and 16%, respectively.

**Table 3:** Distribution of refractive errors according to the age groups.

Age (yr) Type of refraction	Under 30	30–40	Over 40
Emmetropia Refraction mean (D)	997 (73%) 0.20 ± 0.28	720 (68%) 0.19 ± 0.29	126 (69%) 0.22 ± 0.32
Myopia Refraction mean (D)	257 (19%) -2.11 ± 1.61	250 (24%) -2.22 ± 1.72	27 (15%) -2.63 ± 1.89
Hypermetropia Refraction mean (D)	109 (8%) 1.46 ± 1.53	85 (8%) 1.38 ± 1.27	29 (16%) 1.12 ± 0.53
Total Refraction mean (D)	1363 (52%) -0.13 ± 1.32	1055 (41%) -0.28 ± 1.47	182 (7%) -0.08 ± 1.37

D = diopter.

According to type of jobs, subjects were divided into 9 groups. There was no relationship between subject's jobs and refractive errors (*P* = .136) (Table 4).

**Table 4:** Distribution of refractive errors according to the occupational groups.

Occupational groups	Number	Refraction mean (D)
Mining	122 (4.7%)	–0.22 ± 1.11
Metal industry	616 (23.7%)	–0.06 ± 1.78
Detergent industry	125 (4.8%)	–0.11 ± 0.75
Food industry	276 (10.6%)	–0.22 ± 0.96
Chemical industry	273 (10.5%)	–0.08 ± 2.05
Machinery industry	247 (9.5%)	–0.33 ± 1.08
Textile industry	409 (15.7%)	–0.12 ± 0.68
Heavy industry	218 (8.4%)	–0.25 ± 0.82
Others	314 (12.1%)	–0.03 ± 1.98
Total	2600 (100%)	–0.19 ± 1.39

D = diopter.

From total subjects, 164 persons had CVD. Prevalence of color blindness in population under study was 6.3%. Based on objective refraction, 120 (74%) of subjects were emmetropic and 29 persons (18%) were myopic and 13 persons (8%) were hypermetropic (Table 5). Comparison of refractive errors magnitude did not show difference between 2 normal and deficient color vision (*P* = .501). The mean SE of color blind subjects was –0.28 ± 1.63 D with range –9.00 to + 1.50 D. The mean SE of normal color persons was –0.23 ± 1.53 D with range –11.00 to + 10.00 D. In comparison with normal subjects, color vision state had no significant influence on refraction of our subjects (*P* > .05).

**Table 5:** Distribution of refractive errors according to color vision.

Refraction Color vision	Emmetroia	Myopia	Hyperopia	Total
Normal	1723 (71%)	503 (20%)	209 (9%)	2436 (100%)
Abnormal	120 (74%)	30 (18%)	14 (8%)	164 (100%)

## 4 Discussion

Successful performance and safety in working place are related to a good vision in many occupations. Every occupation requires to set vision standards for distance and near vision, color vision, depth perception, and peripheral vision.^[1–3]^ We evaluated 2 important aspects of vision: refractive errors and color vision. Our subjects were workers in the industrial companies. According to our findings, 71% of population under study were emmetropic and had a good visual acuity. However, 29% of our subjects had refractive errors and required to corrective glasses. 20% of subjects were myopic and 9% of them were hyperopic. The number of myopic subjects was more than double that of hypermetropic persons. Fortunately, 24% of our participants had low refractive errors (16% myopia and 8% hypermetropia), but 5% of them suffered from high refractive errors (4% myopia and 1% hypermetropia) that may cause serious visual impairment and need to treat extremely.

Despite our extensive search, we found no study on the prevalence of refractive errors in industrial workers; so, we considered the results of other studies in similar age groups for comparison. In the study by Cortinez^[26]^ on 1518 office-workers, prevalence of myopia and hypermetopia was 29% and 18%, respectively. In the other study by Hashemi et al^[27]^ in 1462 Iranian university students, the prevalence of myopia and hypermetopia was reported 42.71% and 3.75%, respectively. Abdullah et al^[28]^ assessed the prevalence of refractive errors in 1000 adults from a rural area in Pakistan. The prevalence of myopia and hyperopia was 6.0% and 10.1% respectively. Sheeladevi et al^[29]^ in a systematic review assessed the prevalence of refractive errors in adults from various region of India. The prevalence of 27.7% for myopia and 27.7% for hypermetropia was observed. Finally, in the study by Rim et al^[30]^ on 33,355 Korean parsons, the prevalence rates were determined 68.5% for myopia, and 1.2% for hyperopia.

Our findings were different from the results of other studies. The prevalence of refractive errors in our subjects was lower than other studies. These differences may be due to genetic and racial differences, and environmental factors of study populations. Hereditary diseases are more common in some races and have a pattern of familial aggregation. Previous studies have shown a higher prevalence of myopia in East Asian countries and a lower prevalence in Caucasians, which may be due to genetic factors of myopia such as shape and axial length of the eye. Also, myopic parents are more likely to have children with refractive errors and longer axial length of eye.^[16–17,27,31]^ Genetic studies have identified many myopia-associated loci and genetic mutations.^[32]^ Addition to genetic factors, environmental factors and interaction between them should be considered.^[31–33]^The main environmental factors include amount of near work, education, and outdoor activities. In recent years, students in schools and universities have been under educational pressures and have assigned many hours for reading and near work activity. In countries with higher levels of education, all refractive errors, especially myopia was more prevalent. Additionally, use of digital devices has become a routine part of daily life, and many people spend more time in near distance. Studies have been shown increased near work time and decreased outdoor activities were associated with increases in the prevalence and severity of early onset myopia.^[27,30–31,34]^ Interestingly, outdoor activities, as well as natural light were found to have protective effects on the subjects’ refractive errors.^[31,35–37]^It seems that the prevalence rates in our study population is influenced by genetic and environmental factors. Iranian people had lower prevalence rates than East Asian countries. Also, industrial workers spend fewer hours for near work versus persons with jobs involved in near distance and less affected by mechanisms related to near work and myopia development.

Our data showed the prevalence of refractive errors varied in different age groups. The prevalence of refractive errors of subjects in the third and fourth decades were similar, and the prevalence of myopia was more than twice that of hypermetropia. However, in the subjects over 40 years old, the prevalence of hypermetropia increased and became double. The prevalence of myopia also decreased in these subjects. Aging is associated with changes in ocular refraction. Ocular refractive condition becomes stable during early adulthood. After the age of 40, due to reduced accommodation and ocular media changes, an increase in hyperopia occurs.^[38,39]^ Similar results have been reported in other studies.^[26,28,30]^

In our study, the prevalence of color blindness among men was 6.3%. Literature shows prevalence of inherited red-green color deficiency in the world between men 0.8% and 9.3% and women 0.4% and 3.2%.^[22]^ The prevalence of color blindness among Iranian men and women in 1 study by Katibeh et al^[40]^ was reported 4.4% and 0.7%, respectively. The prevalence of CVD in Ethiopia was 4.24%.^[41]^ Khosla et al^[42]^ evaluated the prevalence of color vision anomalies in 100 dental professionals. Of total subjects, 19 reported as having CVD. In other study in India, the prevalence of color blindness was 3.2% among males and 0.00% among females.^[43]^ Despite some differences due to racial and genetic factors, our results were similar to previous studies.

According to our research, refraction of subjects showed no significant relationship with color vision status. Khalaji et al^[20]^ in a study on 1853 subjects with 10 to 25 years old was reported similar results. However 2 other studies stated color blind persons had lower refractive errors. Ostadimoghaddam et al^[44]^ studied on 4400 students. They stated the lower prevalence of myopia and hypermetropia in color blind subjects. Qian et al^[45]^ in other study on 16,539 students reported lower incidence of myopia in color blind people. The fact that there are fewer subjects with a limited range of refractive errors in the color blind group. Also, the age of our research differs from these studies. We performed our study in adults but 2 other study conducted in the children under age 20 years.

CVD causes difficulties in some jobs such as driving, fashion designing, electronic engineering, military, and medicine and education. Some workers may have trouble reading charts and graphs, and lights.^[46,47]^ While most color blind people have likely learned to adapt to their conditions. For patients with difficulties at their jobs, some modalities might help to cope their difficulties and stress. New technologies including gene therapy, tinted glasses, lenses, and advanced features developed on smartphones and computers can enhance the distinction between colors.^[48]^

One of the limitations of this study was that we did not assess the amount of near work and outdoor activities of workers in free times and their impact on the refractive errors. And the other limitation was that we did not use more sensitive color test such as Farnsworth Munsell 100-Hue Test to distinguish between several types of CVD.

## Author contributions

**Conceptualization:** Alireza Akbarzadeh Baghban.

**Data curation:** Masoumeh Ahadi, Afsaneh Ebrahimi, Alireza Akbarzadeh Baghban.

**Investigation:** Masoumeh Ahadi, Afsaneh Ebrahimi.

**Methodology:** Masoumeh Ahadi, Alireza Akbarzadeh Baghban.

**Project administration:** Masoumeh Ahadi.

**Supervision:** Masoumeh Ahadi, Afsaneh Ebrahimi, Saeed Rahmani, Alireza Akbarzadeh Baghban.

**Visualization:** Masoumeh Ahadi, Alireza Akbarzadeh Baghban.

**Writing – original draft:** Saeed Rahmani, Alireza Akbarzadeh Baghban.

**Writing – review & editing:** Saeed Rahmani, Alireza Akbarzadeh Baghban.
